# Visualization of the small RNA transcriptome using seqclusterViz

**DOI:** 10.12688/f1000research.18142.2

**Published:** 2019-06-27

**Authors:** Lorena Pantano, Francisco Pantano, Eulalia Marti, Shannan Ho Sui

**Affiliations:** 1 Biostatistics, Harvard T.H. Chan school of Public Health, Boston, MA, 02115, USA; 2 Independent Researcher, San Juan, Argentina; 3 Biomedicine, University of Barcelona, Barcelona, Barcelona, Spain

**Keywords:** small RNA, miRNA, tRNA, snoRNA, sequencing, visualization, report

## Abstract

The study of small RNAs provides us with a deeper understanding of the complexity of gene regulation within cells. Of the different types of small RNAs, the most important in mammals are miRNA, tRNA fragments and piRNAs. Using small RNA-seq analysis, we can study all small RNA types simultaneously, with the potential to detect novel small RNA types. We describe SeqclusterViz, an interactive HTML-javascript webpage for visualizing small noncoding RNAs (small RNAs) detected by Seqcluster. The SeqclusterViz tool allows users to visualize known and novel small RNA types in model or non-model organisms, and to select small RNA candidates for further validation. SeqclusterViz is divided into three panels: i) query-ready tables showing detected small RNA clusters and their genomic locations, ii) the expression profile over the precursor for all the samples together with RNA secondary structures, and iii) the mostly highly expressed sequences. Here, we show the capabilities of the visualization tool and its validation using human brain samples from patients with Parkinson’s disease.

## Introduction

Small RNAs are 18-36-nt-long RNA molecules that are involved in gene regulation, chromatin structure, and transposon element repression. The most well known small RNAs are miRNAs, endo-siRNAs and piRNAs ^1^. They are typically processed from double-stranded RNA molecules or single-stranded RNA molecules with a hairpin structure ^2^. They bind to members of the Argonaute (AGO) protein family to form the RNA-induced silencing complex that regulates other RNA molecules and plays a key role in gene silencing ^3, 4^. Small RNAs can also regulate chromatin states through histone modification and methylation ^5, 6^. Next generation sequencing technologies have enabled a deeper understanding of miRNAs, and other small RNA types have been detected. For instance, it is now known that miRNA genes generate several mature variants called isomiRs that have been detected in multiple conditions, tissues and species ^7^. Other small RNAs can arise from mature tRNAs (tRNA fragments) or small nucleolar RNAs ^8, 9^. While the biogenesis of these molecules is not well understood, studies suggest that they bind to AGO proteins and perform similar functions ^10, 11^.

High-throughput sequencing is a powerful technique for detecting and quantifying small RNAs. The analysis of small RNA data involves multiple steps for detection, annotation, quantification, and *de novo* discovery of putative small RNA molecules. In general, tools focus on the annotation of known miRNAs ^12^, but new methods to detect other functional types of small RNAs are becoming increasingly important to understand the complex roles of small RNAs. Some tools have been developed to address this challenge ^13– 15^ but few of them produce a visual and interactive report ^16, 17^, and many depend on the use of a remote web server ^18– 21^.

We previously developed seqcluster, a genome-wide small RNA characterization tool that detects units of transcripts (clusters) using a heuristic iterative algorithm to deal with multi-mapped events ^22^. It quantifies all types of small RNAs in non-redundant manner, and extracts patterns of expression in biologically defined groups. This allows us to study any small RNA cluster detected in the samples, including novel regions not previously discovered or small RNAs in species with poorly curated annotations. Here we describe seqclusterViz ^23^, an interactive web-app that reports the output of seqcluster, visualizing small RNA biological features to better understand their putative functions. It allows the user to browse lists of detected small RNAs, shows the precursor secondary structures and the small RNA expression on the precursor, allowing for more in-depth characterization of isomiRs, tRNA fragments, and any other small RNAs detected.

seqcluster and seqclusterViz are integrated into bcbio-nextgen, a community-based Python framework for fully automated high throughput sequencing analysis.

## Methods

### Implementation

seqclusterViz ^23^ is developed in HTML, CSS and JavaScript programming languages. It is a stand-alone tool without external dependencies. It runs locally on one’s computer making it portable and independent. It uses an SQLite JavaScript library to load all the information from a file created by the seqcluster tool ^22^.

### Operation

seqclusterViz ^23^ works on Opera >44.0, Firefox >52.0 and Chrome >57.0. It requires a seqcluster report as input. An Internet connection is not required. The tool can be downloaded from its home page ( https://github.com/lpantano/seqclusterViz/archive/master.zip). After extracting the ZIP file content, the user can open the index.html file with the desired web browser. The user first clicks the ’UPLOAD’ button and then selects the seqcluster.db file. Once the data has been uploaded, the top-left panel displays all of the small RNA transcripts detected. Each small RNA transcript is clickable to obtain more information ( 1A). After selecting a small RNA transcript, the top-right panel shows the genomic locations for that transcript. The middle-left panel displays the abundance profile along the precursor ( 1B); the middle-right displays the RNA secondary structure ( 1B); as calculated by seqcluster with RNAfold and default parameters ^24^; and the bottom table shows the top 50 most abundant sequences. This table can be sorted and searched using text queries ( 1C).

**Figure 1.**
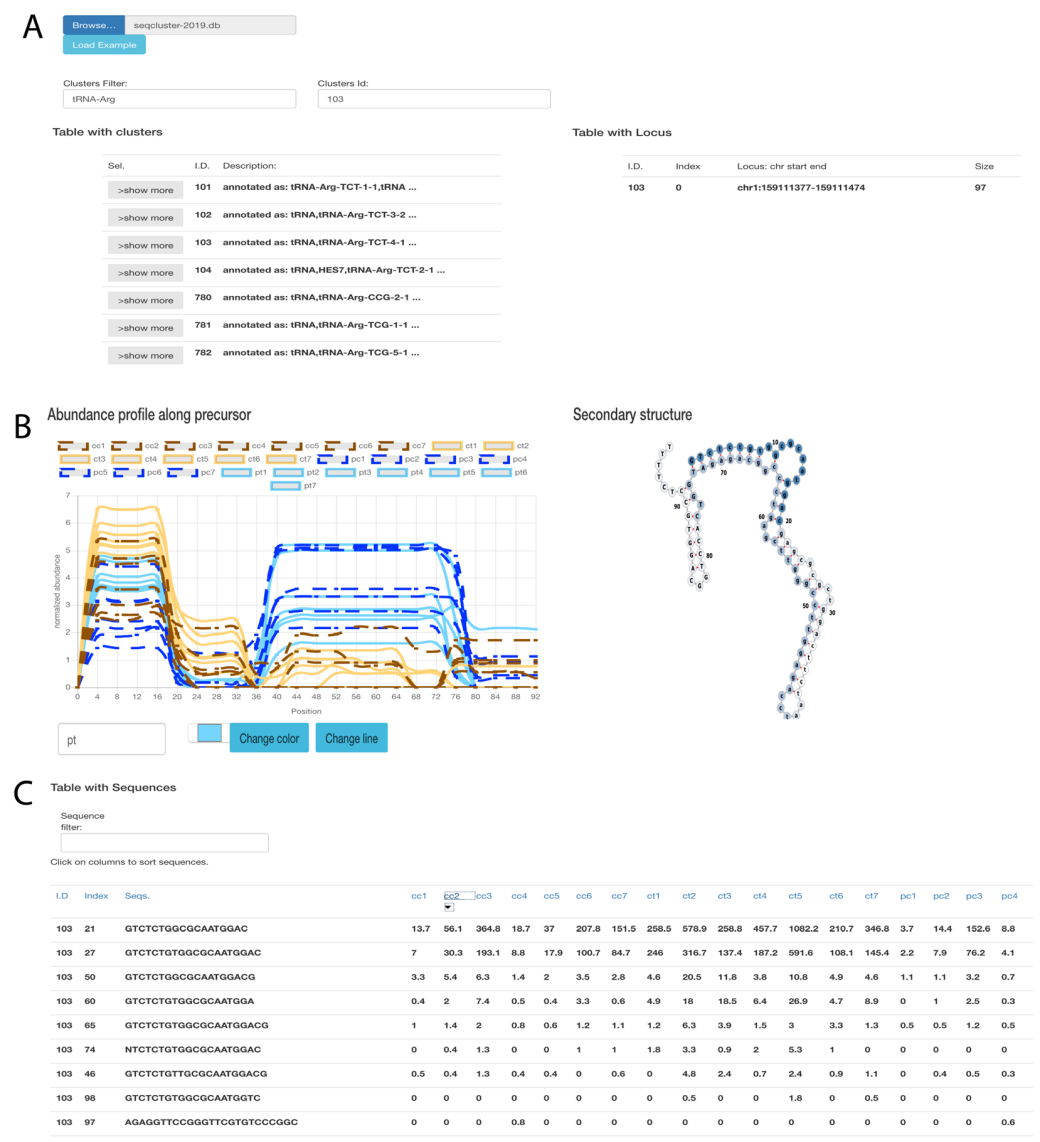
seqclusterViz features. ( **A**) Top panel with table showing the list of small RNAs detected (left) and genomic location (right). ( **B**) Middle panel shows abundance profile over the precursor (left), and secondary structure (right). This is an example of batch effect at the 3’ end (blue higher than brown) and disease effect at the 5’ end (solid lines higher than dashed lines). ( **C**) Bottom panel shows a table with the top most expressed sequences on the selected small RNA transcript. The index column is the sequence identifier that links the results to the original seqcluster output files.

The tool provides a number of formatting options to emphasize differences between groups and/or samples and to customize figures. Figures can be exported by right-clicking on it. This provides an easy and quick option to generate publication-ready material.

## Use cases

We used public data from 14 human brain samples at pre-motor (PT) and motor (CT) stages of Parkinson’s disease (GEO accession number GSE97285) and 14 healthy human brain samples (pre-motor controls - PC and motor stages control - CC) ^22^. Data was analyzed with bcbio-nextgen using piDNA to detect the adapter ^25^, cutadapt to remove it ^26^, STAR to align against the hg19 genome assembly ^27^, and seqcluster to detect small RNA transcripts ^22^. We used the output seqcluster.db from *seqcluster report* command to test seqclusterViz ^23^. It took four seconds to upload this 28 MB file to the web page. This dataset is affected by a batch effect for the two Parkinson’s groups due to the groups being sequenced at different read lengths. PC and CC samples were derived from the same RNA extraction, and were expected to show similar expression profiles. However, there is a clear difference by batch (brown versus blue) that is visually apparent in the abundance profile of the tRNA-Arg-TCT RNA across the length of the transcript in ( 1B). Longer reads allow for detection of longer small RNAs since the 3’ adapter can be recognized during the analysis (there is a requirement to include adapter sequences in the seqcluster tool). The longer reads from the PC/PT samples (blue) permitted detection of longer small RNAs at the end of the precursor, generating the batch difference in the abundance profile. Moreover, there is a difference in expression at the 5’ end of the precursor, where Parkinson’s samples (solid lines) are higher than their respective controls (dashed lines). The secondary structure of this small RNA shows a pre-miRNA-like hairpin structure (with a stem-bulge-stem and a terminal-loop) that is normally required to be processed into 18-33-nt mature molecules, where the stem-bulge-stem section encodes the mature sequence ^28, 29^. Although the structure is larger than typical pre-miRNAs, it is still possible to process with the miRNA machinery. Thus the secondary structure of the molecule can serve as an additional feature to evaluate when seeking candidates for further experimental validation. Quantitative polymerase chain reaction (qPCR) or small RNA transfection technologies are often used to validate small RNA stability and function. To do so, a single small RNA needs to be used as the target sequence for these assays. The table at the bottom of the page ( 1C) allows users to select the most abundant sequence in the current small RNA that can be used for such experiments.

## Summary

seqclusterViz ^23^ helps users to explore the expression profiles of detected small RNAs across the length of the precursor, the secondary structure of the small RNA, and the annotation. We show the importance of visualizing small RNAseq data to prioritize candidate small RNAs for further experimental validation or functional analysis. The user can modify the figure format and export it for publication or presentation purposes. It is also possible to select the most highly expressed sequence of a transcript cluster that can be used for qPCR or for cell transfection assays.

## Data availability

Data to reproduce this analysis is available from the Parkinson project page.

Data from 14 healthy human brain samples were originally reported by Pantano *et al.* ^22^. Data from 14 human brain samples at pre-motor (PT) and motor (CT) stages of Parkinson’s disease are available at GEO, accession number GSE97285.

The web-tool can be tested at GitHub pages. Click on **Load Example** to start using the tool with the example data set.

## Software availability

seqclusterViz **is downloaded from:** https://github.com/lpantano/seqclusterViz/archive/v0.1.2.zip.

**Source code available from:** https://github.com/lpantano/seqclusterViz.

**Link to source code as at time of publication:** url https://doi.org/10.5281/zenodo.3250205 ^23^.

**License:** MIT License.
