# Supplementary material for: Visualization of the small RNA transcriptome using seqclusterViz
Source: F1000Res. 2019 Jun 27;8:ISCB Comm J-232. Originally published 2019 Feb 28. [Version 2] doi: 10.12688/f1000research.18142.2 (PMC6446497; doi:10.12688/f1000research.18142.2)
Supplement: Peer review history

# Peer review: Reviewer response for version 1

**DOI:** 10.5256/f1000research.19842.r45091

**Published:** 2019-04-01

**Authors:** Stefan Scholten

**Affiliations:** 1 Crop Plant Genetics, Georg-August-University Göttingen, Göttingen, Germany

The article reports on a visualization tool for seqcluster outputs, a software tool to characterise small transcriptome data. It is a smart tool for sRNA visualization. The interactive view makes it attractive, especially the visualization of secondary structures. The performance of the tool fits description and the filter option is very helpful for jumping to the desired information.

 The tool is restricted to seqcluster.db file as input and cannot be used as a general-purpose sRNA visulization tool using map files.

 Sufficient information is provided to allow interpretation of the expected output data sets and results generated with the tool. The example expression profile provides clear distinction between samples.

 The editing option makes it even better, but when one switches to the line view different lengths can be seen. It is not clear whether this is related with the length of the sRNA that map at that position. A summary of the lengths that mapped would be useful additional information. Alongside the description section in Figure A (left side) information on the length of sRNA should be included.

I confirm that I have read this submission and believe that I have an appropriate level of expertise to confirm that it is of an acceptable scientific standard.

# Response

**Published:** 2019-04-10

**Authors:** Lorena Pantano Rubino

**Affiliations:** Harvard TH Chan School, USA

Dear Stefan Scholten,

Thanks for finding time to review this article. I will work on your recommendation to add that information to the tool, it is a very good idea we missed.

I'll post back the update version here.

Cheers

# Peer review: Reviewer response for version 1

**DOI:** 10.5256/f1000research.19842.r45803

**Published:** 2019-03-18

**Authors:** Xavier Bofill-De Ros

**Affiliations:** 1 RNA Mediated Gene Regulation Section, RNA Biology Laboratory, Center for Cancer Research, National Cancer Institute, Frederick, MD, USA; 2 National Cancer Institute, Frederick, USA

This article by *Pantano et al.* describes a novel software, SeqclusterViz, to visualize and help the interpretation of small RNA-seq data mapped using Seqcluster. Moreover, the authors illustrate its usage with a case study of small RNAs dysregulated in the brain of patients with Parkinson’s disease. This software is of importance to help researchers do in depth analysis and compare the exact mapping of reads across multiple samples. The subject of this manuscript is interesting and seems adequate for indexing in F1000Research. The work looks solid although minor edits could be performed to improve the quality and usability of the SeqclusterViz.

**Minor comments:**

- The SeqclusterViz displays the sequence of the reads mapped to a precursor RNA such as a pri-miRNA. In particular, mature miRNA is well known for having isoforms (isomiRs) with the addition or internal edit to non-templated nucleotides. The field of isomiR study is of growing interest, SeqclusterViz will benefit from a display of non-templated nucleotides.
- A secondary structure prediction is implemented on SeqclusterViz, please describe in more the prediction method used. Include input parameters (such as if GU pairs at the end of helices) and outputs such as MFE, bracket-dot notation...
- Provide units for the Y and X axis in “Abundance profile along precursor”.
- Provide normalized read counts on “Table with Sequences”.
- Repair the path to make the button “Load example” active.
- The buttons of “Add filter” and “Change line” can’t be linked to the samples easily.

I confirm that I have read this submission and believe that I have an appropriate level of expertise to confirm that it is of an acceptable scientific standard.

# Response

**Published:** 2019-04-10

**Authors:** Lorena Pantano Rubino

**Affiliations:** Harvard TH Chan School, USA

Dear Xavier Bofill-De Ros,

Thanks a lot to find time to review this article. I will take actions on all the points I can address in the next month and report an update as soon as possible.

Cheers
